# Addressing disparities in European cancer outcomes: a qualitative study Protocol of the BEACON project

**DOI:** 10.3389/fpsyg.2024.1252832

**Published:** 2024-02-26

**Authors:** Giulia Ferraris, Veronica Coppini, Dario Monzani, Roberto Grasso, Iva Kirac, Denis Horgan, Ricardo Pietrobon, Victor Galvão, Gabriella Pravettoni

**Affiliations:** ^1^Applied Research Division for Cognitive and Psychological Science, IEO, European Institute of Oncology IRCCS, Milan, Italy; ^2^Department of Psychology, Educational Science and Human Movement (SPPEFF), University of Palermo, Palermo, Italy; ^3^Department of Oncology and Hemato-Oncology, University of Milan, Milan, Italy; ^4^Genetic Counseling Unit, University Hospital for Tumors, Sestre Milosrdnice University Hospital Center, Zagreb, Croatia; ^5^European Alliance for Personalized Medicine, Maribor, Slovenia; ^6^SporeData OÜ, Tallinn, Estonia

**Keywords:** cancer disparities, healthcare divide, digital divide, accessibility, health literacy, cancer inequity, cancer inequality

## Abstract

**Introduction:**

Health disparities represent a crucial factor in cancer survival rates, awareness, quality of life, and mental health of people receiving a cancer diagnosis and their families. Income, education, geographic location, and ethnicity are some of the most important underlying reasons for health disparities in cancer across Europe. Costs of healthcare, access to information, psycho-oncological support options, integration of cancer research and innovative care, and multidisciplinary cancer teams are the main target areas when it comes to addressing disparities in the cancer context. As part of the Beacon Project (BEACON), we developed a protocol for a qualitative study to explore and identify any relevant reasons for cancer inequalities and disparities in Europe.

**Methods:**

Our four stakeholders namely, cancer patients, healthcare providers, researchers, and policymakers will be recruited online, facilitated by collaborative efforts with cancer organizations from various European countries, including but not limited to Italy, Croatia, Estonia, and Slovenia. Qualitative online focus group discussions for each stakeholder will be conducted and transcribed. Subsequently, thematic analysis will be used to identify reasons and aspects that may contribute to the existing disparities in cancer outcomes at various levels of engagement and from different stakeholders’ perspectives. Results from focus groups will inform a subsequent Delphi study and a SWOT analysis methodology.

**Discussion:**

Although advances in medical research, cancer screening and treatment options are constantly progressing, disparities in access to and awareness of healthcare in cancer patients are even more noticeable. Thus, mapping the capacity and capability of cancer centres in the European Union, creating decision support tools that will assist the four stakeholders’ information needs and improving the quality of European cancer centres will be the main objectives of the BEACON project. The current protocol will outline the methodological and practical procedures to conduct online focus group discussions with different stakeholders.

## 1 Introduction

In the last decades, advances in cancer therapy and research have led to a greater awareness of the existing gap in cancer outcomes (e.g., diagnosis, treatment, management of survivorship, quality of life, palliative care) among the world population. Access to treatment, survival rates, and cancer recognition have improved for the better-educated and wealthier population. Also, people belonging to ethnic majorities, living in urban areas and having access to the Internet have benefited from the general progress in cancer research (Hendren et al., 2010). However, there are salient disparities in cancer outcomes, within and between European countries, often associated with race, income, education, geographic location and access to the Internet (Gross et al., 2008; Ferraris et al., 2023). The EU-funded BEACON project aims at detecting and addressing underlying factors contributing to cancer disparities from the direct perspective of four different stakeholders, primarily the patients and, subsequently, healthcare providers, researchers and health policymakers.

In Europe, differences in cancer survival rates between European nations are significant, as well as variations within the countries themselves (De Angelis et al., 2014). The situation is quite serious in Eastern Europe, where mortality rates are higher than the European average for various cancer types (European Cancer Patient Coalition, 2015). For instance, mortality rates for lung cancer in Europe are at the highest in Hungary (78–88.3 per 100,000 people) and Poland (67.8–78.0 per 100,000 people) while mortality rates for stomach cancer appear to be greater (18.2–21.4 per 100,000 people) in Estonia, Latvia, and Lithuania (European Cancer Information System, 2022). Severe disparities in cancer outcomes are also evident in Croatia, where cancer mortality is the second highest in the EU, after Hungary, with 25% of mortality higher than average (European Cancer Inequalities Registry, 2023). Disparities are also relevant in Italy, while cancer incidence and mortality are lower than the EU average, there are significant inequalities on a regional level regarding the management of risk factors, screening programs and participation (European Cancer Inequalities Registry, 2023). Notwithstanding the variations both between and within countries, the most conspicuous disparities across Europe remain evident in several critical aspects of cancer outcomes. These encompass cancer screening, diagnosis, treatment, quality of life, survivorship management, and end-of-life palliative care in numerous European nations. At the root of such disparities are socioeconomic, educational, demographic and digital inequalities. Specifically, at the top of the list there are difficulties in accessing updated and reliable health information (Warren et al., 2014), inconsistency in cancer screening programs and services (Elfström et al., 2015), challenges in accessing treatment and rehabilitation services (Baili et al., 2013; Meara et al., 2015), inadequate enrolment in clinical trials (Denicoff et al., 2013), lack of personalized and regionally attuned approach to support the well-being and quality of life of cancer survivors in Europe, and several deficits in health planning (Lawler et al., 2013; Coleman and Allemani, 2015). Gaining a better understanding of the reasons exacerbating these disparities in access to care and the quality of care for cancer patients becomes essential because these disparities can have a significant impact on their outcomes, including survival rates and quality of life. Among factors contributing to cancer disparities, learning and having access to health-related information and sources (e.g., Internet, healthcare providers, etc.) that are up to date and accurate, is an important factor in reducing anxiety and distress in cancer patients, decreasing the rate of disinformation and indecisiveness about treatment and influencing treatment adherence (Jenkins et al., 2001; Warren et al., 2014). Previous research has indicated that cancer patients often lack awareness of the best healthcare professionals available near their residences and frequently require improved information and support. The provision of appropriate information regarding cancer screening, diagnosis, treatment, and quality of life is crucial to assist patients in navigating the entire continuum of cancer. However, in many instances, the quality of the provided information is excessively intricate and inaccessible to the average reader, resulting in heightened disparities (Lange et al., 2017). Perceptions of inequalities and disparities in cancer outcomes can also be intensified by interpersonal factors, such as the doctor-patient relationship. Some studies have highlighted a significant challenge for cancer patients in communicating their needs and preferences to clinical staff, which, in turn, may inadvertently lead healthcare professionals to overlook their patients’ needs (Gonzales et al., 2019). Thus, obtaining opinions, including experiences, needs and preferences on the matter directly from cancer patients themselves would be a critical factor to consider while addressing cancer disparities.

Furthermore, disparities are also observed among nations in terms of how healthcare providers organize and define a “care pathway” for the standard process from diagnosis to treatment (Blay et al., 2021). In certain European countries, cancer screening programs are either nonexistent or minimally communicated to the public (Lynge et al., 2012). Cancer mortality rates, which are clearly the major issue, have shown significant falls after the implementation of organised screening programs and services (Quinn et al., 1999). In particular, such decreases in cancer mortality rates and incidence have been observed in England, Italy, Finland and the Netherlands (Dyba et al., 2021; Cazzolla Gatti et al., 2022). Moreover, surgical procedures, individualised treatment, and rehabilitation services need to be widely spread and available in order to reduce the risk of cancer and mortality in cancer patients (Meara et al., 2015). To enhance awareness, the promotion of screening and cancer treatment information should adhere to evidence-based practices. For instance, Germany has recently adopted a model of “informed participatory decision-making” to enhance patient information, train healthcare professionals in effective communication, and elevate public health awareness (Gigerenzer, 2015). The German model could provide a valuable illustration, especially in educating healthcare providers on informing and empowering patients. Nonetheless, this transition requires recognizing the right to knowledge and it presents the dilemma that well-informed patients may not always result in higher engagement in screening programs. Hence, it would be advantageous to comprehend, from the viewpoints of both patients and healthcare providers, the factors contributing to the high level of information and compliance among patients, with the aim of addressing cancer disparities.

Another crucial aspect when it comes to the implementation of new treatments, advances, and solutions in oncology is that of clinical trials, but, unfortunately, several barriers are encountered when it comes to enrolment and putting in place the research questions in clinical trials (Denicoff et al., 2013). In order to address the aspects of cancer outcomes related to the disadvantaged population, there should be specific and targeted enrolment procedures that take into account the reasons for the disparities in clinical trial participation at patient/community, physician/provider and site/organisational levels (Mills et al., 2006; Denicoff et al., 2013). Major reasons for disparities in clinical trial participation are older age, belonging to an ethnic minority and male gender (Stewart et al., 2007). Thus, collaboration between researchers and healthcare providers in relation to planning more targeted clinical trials is needed. Actually, it has been a primary concern for the World Health Assembly, for decades, to set objectives and targets in order to improve accessibility to screening, treatment and care in the cancer patient population. In addition, the World Oncology Forum, in 2012, appointed the need for new strategies aimed at prevention and more successful and affordable treatment (World Oncology Forum Writing Committee, 2013; Coleman and Allemani, 2015).

Finally, policymakers lack information about patients’ needs, thus creating a misdirection regarding where funds should be invested and difficulties in integrating patient perspectives into the policymaking process. Existing literature suggests that aligning policies with patients’ needs may be linked to reduced death rates in cancer. For instance, numerous northern and western European countries have seen a notable decline in colorectal and lung cancer rates through health policies addressing food consumption, smoking awareness campaigns, improved water sanitation, lifestyle adjustments, and enhanced environmental conditions (Bertuccio et al., 2019). Lower death rates in lung and bladder cancer are also attributed to improved working conditions and reduced exposure to occupational carcinogens (Negri and Vecchia, 2001; Antoni et al., 2017). However, these policies are not universally implemented, and they may not always be tailored to meet patients’ needs, as seen in Eastern European countries like Romania, Russia, and Ukraine. In these regions, lack of policies, delays in adopting effective screening programs and treatments coincide with higher mortality rates, especially for breast and lung cancer (Levi et al., 2004; Bertuccio et al., 2019). Hence, the collaboration between policymakers, researchers, and healthcare providers is crucial for resource allocation and the implementation of guidelines and health policies aligned with individual patient priorities. This approach aims to reduce cancer risk and enhance awareness across diverse countries.

Although the topic of cancer disparities is conspicuously discussed and brought to attention in the literature (Mills et al., 2006; Stewart et al., 2007), little research is suggesting concrete and broad-spectrum solutions that address different stakeholders including patients, healthcare providers, researchers and policy makers. Enhancing accessibility to Internet sources, information, healthcare providers (Ferraris et al., 2023), screening programs and treatment is a crucial aspect to consider when aiming at reducing cancer disparities. Understanding the needs and preferences as well as barriers and facilitators of the above mentioned cancer stakeholders plays a pivotal role in affecting disparities in cancer outcomes, impacting various aspects such as treatment adherence, access to care, quality of life, patient-provider relationships, and psychosocial support. By prioritizing these factors in cancer outcomes, we can make strides in reducing disparities and enhancing the overall quality of life and survival rates for individuals grappling with cancer.

Therefore, the present study proposes to address the informational, treatment and support needs of cancer patients, healthcare providers, researchers and policymakers as well as to identify potential barriers in the cancer context in order to tackle the issue of the cancer divide in its entirety.

### 1.1 Primary objective

The current protocol describes the procedure and the methodology of online focus group discussions with cancer patients, healthcare providers, researchers and policy-makers from different European countries, including (but not limited to) Italy, Estonia, Slovenia, and Croatia, which are the four countries to which the partners of the BEACON project consortium belong to. The primary objective of the focus group discussions is to uncover the underlying causes and key factors contributing to disparities and inequalities in cancer outcomes, with a particular emphasis on barriers and facilitators related to access to care, treatment, psychological support, information-seeking behaviors, and decision-making processes. Also, this study seeks to explore potential solutions that can support stakeholders in meeting their needs and preferences concerning disparities and inequalities in cancer outcomes.

### 1.2 Secondary objective

This qualitative study is conducted as part of the BEACON project that will create a website named “BEACON Wiki” to allow the stakeholders to search, explore, evaluate, revise, generate, and update cancer information. The Wiki repository will store all public information about Cancer Centres and their countries and will be displayed in all official EU languages. In the project’s second phase, information from the Wiki will be used to create a decision-support application with personalised interfaces for each stakeholder. For example, these tools might include resources for aiding patients in making informed decisions about the most suitable facilities for their care needs, supporting healthcare providers with education materials and guidelines for healthcare providers, assisting researchers in identifying appropriate hospitals for conducting future clinical trials, and serving policymakers by collecting information on policymaking initiatives and reports crucial for informed decision-making in cancer healthcare policy. Lastly, the current project is aimed at improving the quality of European cancer centres by ensuring they stay updated on the latest advancements in care, thus enhancing the overall quality of services provided.

BEACON consortium comprises a team of psychologists, oncologists, data scientists and policymakers from different countries: European Institute of Oncology (IEO; Italy), SporeData (SD; Estonia), University of Palermo (UNIPA; Italy), the European Alliance for Personalised Medicine (EAPM; Slovenia), and the Klinicki bolnicki centar sestre Milosrdnice ustanova (SMUHC; Croatia). This project is aimed at creating a comprehensive and sustainable model for governance and policy by gathering information on cancer disparities from different stakeholders. The mapping of EU cancer treatment capacity and capability in the European countries is expected to result in facilitating the delivery of higher-quality care and reduce inequalities.

## 2 Methods and analysis

### 2.1 Study design: focus group discussions

This qualitative study will consist of at least 20 focus group discussions:

- At least 5 focus groups (1a, 1b, 1c, 1d, 1e) involving cancer patients, if they are not available for the discussion informal caregivers (i.e., family members or friends providing care to cancer patients) could participate on their behalf.
- At least 5 focus groups (2a, 2b, 2c, 2d, 2e) involving healthcare providers of hospitals and cancer centres in the European Union.
- At least 5 focus groups (3a, 3b, 3c, 3d, 3e) involving researchers and scholars working in the oncology research field.
- At least 5 focus groups (4a, 4b, 4c, 4d, 4e) involving policymakers (i.e., people of government or decision-making institutions, including international organisations, non-governmental agencies or professional associations).

Qualitative methods, including focus groups, offer a valuable means to explore complex issues like cancer disparities comprehensively. These disparities involve multifaceted factors such as cultural, socioeconomic, and institutional influences. Focus groups enable participants to openly discuss these factors, providing a nuanced understanding, as qualitative research goes beyond just “what” to uncover “why” and “how” disparities exist (Krueger R, 2002). Employing a focus group design can be viewed as a crucial initial phase in the development of digital decision support tools, which is the ultimate goal of the BEACON project. This approach holds significance because it enables direct interaction with prospective users of the decision support tools, encompassing healthcare providers, patients, and policymakers alike. This user-centric methodology guarantees that the tools are meticulously crafted to align with the precise requirements and preferences of the intended audience (Sanz et al., 2021).

**Figure 1:**
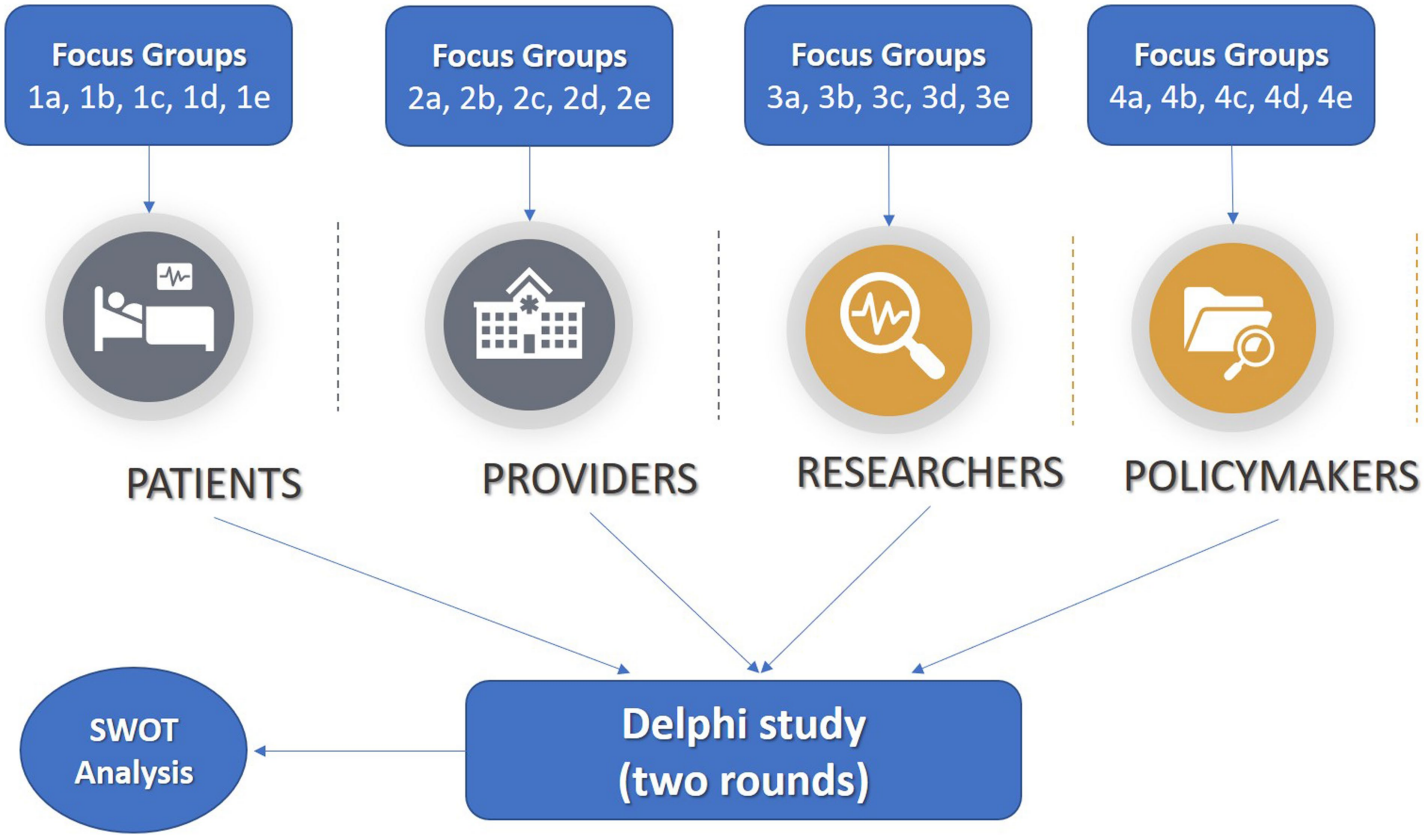
Fully proposed study methodology with arrows indicating study methodology and target population. Focus groups (1a, 1b, 1c, 1d, 1e) will be conducted with cancer patients. Focus groups (2a, 2b, 2c, 2d, 2e) will be conducted with healthcare providers. Focus groups (3a, 3b, 3c, 3d, 3e) will be conducted with researchers. Focus groups (4a, 4b, 4c, 4d, 4e) will be conducted with policymakers. Findings from the focus groups will inform a Delphi study and a SWOT Analysis with patients, healthcare providers, researchers and policymakers.

While focus groups offer rich insights, there are potential pitfalls to consider. Group dynamics might lead to dominant voices overshadowing quieter participants or individuals withholding their opinions due to social pressures. Additionally, the sample size and composition could impact the breadth of perspectives represented, although we followed guidelines for data saturation (Guest et al., 2006). Regarding online recruitment methods, there are limitations as well. These approaches might inadvertently exclude certain demographics, especially those with limited internet access or technological proficiency, potentially skewing the participant pool. Balancing these limitations requires careful consideration, possibly employing diverse recruitment strategies to ensure a more comprehensive representation of perspectives.Results from focus groups will inform a subsequent Delphi study and a SWOT (Strengths, Weaknesses, Opportunities, Threats) analysis methodology. For the fully proposed study methodology see Figure 1.

### 2.2 Setting

All the participants will be recruited across centres situated in the European countries taking part in the BEACON project consortium including Italy, Slovenia, Estonia, and Croatia with the possibility to extend the study’s population to other countries such as Portugal, Spain, France and the UK. The focus group discussions will be held online through Microsoft Teams, for ease, timing and economic reasons.

### 2.3 Data collection

After obtaining the informed consent form and a brief self-report questionnaire, including socio-demographic information, each focus group discussion will be organised and held separately on a specific date and time. All the focus group discussions will be audio-video recorded. The transcripts of the discussions will then be adjusted removing any personal information regarding the participants and then translated into English in order to be analysed.

The current study will include a minimum of 6 and a maximum of 8 subjects for each of the approximately 20 focus groups; hence the number of participants will be at least 30 for each of the four stakeholders (patients, healthcare providers, researchers, and policy makers), making a total of 120 subjects, at the minimum, participating in this focus group study. These sample numerosities were determined according to relevant guidelines (Krueger R, 2002), but the final number of the focus groups will be also determined by considering data saturation. Data saturation is defined as “the point at which no new information or themes are observed in the data” (Guest et al., 2006); moreover, “this redundancy signals to researchers that data collection may cease” (Guest et al., 2006). According to Hennink and colleagues (Hennink et al., 2019), study purpose could be considered as a parameter influencing data saturation and, specifically, if the aim concerns identifying core issues in data, fewer focus groups are needed. Others considered that the shared rule is to plan three or four focus groups for each type or category of individual (Krueger R, 2002). As our aim is to identify core relevant reasons for disparities, we expect to reach saturation with 5 focus groups for each stakeholder group. If saturation is not reached after the planned number of focus groups, other focus groups will be planned until data saturation. To determine data saturation, we plan to implement several strategies. For instance, the evaluation of information redundancy in focus group discussions. This redundancy emerges when successive data collection yields repetitive information without introducing new insights. Thematic analysis presents itself as an effective method to oversee and authenticate saturation, particularly in the analysis of transcribed data. Saturation becomes apparent during thematic analysis as established themes persistently reappear, accompanied by a diminishing emergence of new themes. Moreover, saturation might be determined following an exhaustive exploration of diverse participant perspectives, experiences, or demographic elements, denoting the absence of significantly new information. Additionally, seeking validation through discussions with co-authors serves as a reinforcing method to confirm the occurrence of data saturation. This multifaceted approach enhances the comprehensiveness and rigour of the study’s outcomes, ensuring a robust comprehension of the findings.

To be included in the study and participate in the focus group discussion, individuals must meet all the following eligibility criteria:

- being 18 years or older;
- being able to understand and speak Italian or Croatian or Slovenian or Estonian;
- having access to the internet;
- subjects will be excluded if one or more of the following exclusion criteria is met:
- inability to fully understand written or spoken information;
- a cognitive impairment that would compromise the participation;
- psychological distress that would make the participation an excessive burden.

In addition to the above-mentioned criteria, there are further inclusion criteria for enrolment that are stakeholder specific:

- Patients must have a cancer diagnosis of any type. If not available, their informal caregivers can participate. Caregivers’ experiences might offer valuable insights into patient challenges and disparities, contributing to policy discussions and support initiatives. This inclusion enhances the depth of our data and aligns with patient-centred research principles, particularly in palliative care contexts.
- Healthcare providers must work or be specialised in relation to cancer diseases (i.e., medical oncologists, radiation oncologists, surgeons, clinical oncologists, psycho-oncologists involved in the treatment, palliative care specialists, physical and rehabilitative medicine specialists); Providers must also demonstrate a 5-year experience, at least, in cancer treatment.
- Researchers must be participating in cancer related research and have at least 5 years of experience as researchers in the oncology field.
- Policy makers are people of the government or decision-making institutions, including international organisations, non-governmental agencies or professional associations scholars who have responsibility for making cancer-related recommendations to others; also, they need to have a 5-year experience in cancer related policy-making.

The study design follows a risk minimization and a benefit maximisation requirement, thus promoting non-maleficence and active beneficence towards our participants. Whilst, we do not envisage the risk of participation to be high, potential risks are continuously monitored and classified according to their likelihood and severity of impact on the project and mitigation actions are being assigned accordingly. Participants will participate in a qualitative study that will take a reasonable amount of their time. Whilst not particularly burdensome, there is a possibility that some participants will experience mild distress. To help mitigate this risk, participants can withdraw from the study at any time. In regard to the researchers, no risks have been identified for the conductance of the study. Lastly, it will be made clear in the Information letter that participants can leave the study at any time for any reason if they wish to do so without any consequences. Participants do not receive monetary incentives but there are potential long-term benefits to others and thus indirectly participants may derive satisfaction from this. The information provided in this study will help to identify and mitigate cancer disparities in Europe.

### 2.4 Recruitment methods

Two different approaches of recruitment will be implemented in the present study:

1. Recruitment through broad-based, community, postal and social media, including online advertising on the BEACON project’s website;
2. A more targeted recruitment approach through patient associations, patient advocacies, scientific societies, and direct contact with people within the BEACON project’s consortium.

In order to reach the target population for the study, a multi-language advertisement/flyer will be released, in both cases, inviting adults who meet the above listed inclusion criteria to participate in the study. Participants will be contacted by the researchers via phone call or email and will be given a detailed explanation of the study procedures and duration, together with the time and date of the focus group discussion they would have to attend. To ensure the representation of diverse ethnic and socio-economic backgrounds in our study, we will engage associations from different regions and countries and, before conducting the online focus groups, we will administer a concise socio-demographic questionnaire that explicitly collects data on ethnicity and socio-economic status. Eligible participants will then receive the informed consent form through an *ad-hoc* online template and asked to fill and sign it. In the information letter, it will be stated very clearly that participants are free to leave the study at any time for any reason without any consequences.

### 2.5 Focus group scheme

The duration of the focus group discussions will be approximately 90 min, comprising, if necessary, a 5-min break in between to avoid cognitive burden and tiredness of the participants. The focus group discussions will be differentiated based on the stakeholder. More information regarding each focus group scheme are reported in the Supplementary material. During the focus group discussions, two researchers will be present, one acting as the moderator and the second one will be taking notes and assisting the moderator in the conduction of the discussion. All interviews will be recorded using both the native and an external software to ensure that recordings are reliable. The questions will all be open and the directing of the responses will be avoided, as a matter of fact, the focus will be on broad themes of interest to the project. The interviewers will be matched to the participants with regard to language, in fact the discussions will be conducted in the native language of the attendees to ensure a more accurate understanding of questions and expressing of thoughts and personal opinions. At the end of the focus group, participants will be debriefed and greeted. The audio recording files will be transcribed, removing any personal information of the participants, and then translated into English for analysis.

### 2.6 Delphi study

Participants will be chosen based on their willingness to participate and knowledge of the topic. A heterogeneous group including experts (~ 10/15 experts) with knowledge of specific relevant themes related to cancer disparities will be created. Panel members will comprise oncologists, hospital administrators, policymakers, regulatory experts in data safety and security, cancer patients and their informal caregivers from different countries (e.g., Italy, Slovenia, Croatia, Estonia). The Delphi exercise will involve a series of mailed surveys with questions focused on the most prevalent types of cancer (e.g., treatment, quality of life for survivors, and palliative care), the different stages in cancer, underlying reasons for disparities (e.g., geographic location, education level, immigrant status, religious groups, and language barriers), and research priorities (Shariff, 2015). The mailed surveys will be translated into the native language of the participants (e.g., Italian, Estonian, Slovenian, Croatian and other potential additional languages Portuguese, Spanish, English, and French) by each institution.

Two rounds are expected. More than two rounds increase panel attrition, so this is not recommended.

- The first-round questionnaire will present a series of statements that the respondent is asked to rate on a clearly defined 1–9 Likert Scale (1 = lowest and 9 = higher ratings). The content of the statements will come from a variety of sources, including previous research findings from the focus groups and the literature. Participants will be asked both to rate the item and to write free-text comments that, for example, explain their rating or express disagreement with the statement’s relevance.
- The responses to the first-round questionnaires are collated and used to create the second-round questionnaire.
- Responses will be summarized between rounds and communicated back to the participants through a process of controlled feedback (i.e., group feedback to panel respondents). This process is repeated until consensus is reached.
- Reminders will be sent to non-responders.
- We will also select members for a Steering Committee from our team of investigators. The Steering Committee will summarize responses from the iterative Delphi consensus rounds, prepare group feedback to panel respondents, and identify any concerns moving forward to reaching consensus. For example, if the panel cannot agree on a specific characteristic of the cancer therapy, such as the palliative care at the end-of-life, then the steering committee might make this decision based on the input from all panel members. Although the Delphi consensus technique intends to allow panel members to judge and filter the provided information, the Steering Committee may need to make some decisions to reduce the number of protocols if the panel cannot achieve a consensus on many criteria. This approach will be necessary to prevent the risk of overburdening Delphi panelists for subsequent rounds.
- Consensus will be reached if the agreement rate is higher in the second round (75% or higher). If agreement is reached among panel members after the second round, the Delphi procedure will end. When reaching a consensus will be difficult or unclear, the Steering Committee will stop the process and select a set of protocols from the remaining ones.

### 2.7 SWOT analysis

A SWOT (Strengths, Weaknesses, Opportunities, and Threats) strategic planning analysis will be obtained from the results of content analyses of the focus groups and Delphi surveys aiming to assist Beacon planning to both evaluate and address cancer disparities.

To do so, a scientific approach named SWOT analysis will be used in order to identify:

1. Strengths, which are the organizational characteristics that can help achieve the desired outcome;
2. Weaknesses, which are organizational characteristics that may be harmful to achieving the outcome;
3. Opportunities, or the helpful conditions outside of the organization to achieving the outcome;
4. Threats, which are the harmful conditions outside of the organization to achieving the outcome.

As examples for the four categories, we can highlight:

- Strengths: patient engagement and a combined effort of patients, clinicians, hospital administrators, patient advocacy groups, policymakers, IT industries, the research community, and medical society.
- Threats: data privacy, and security breaches.
- Opportunities: all of the stakeholders (i.e., patients, healthcare providers, researchers and policymakers) lack curated, high-quality data along with training and learning resources; this project will address this gap. There is a wealth of data from diverse populations that are currently scattered, including databases, registries, and guidelines regarding cancer outcomes. Currently, these resources are not being leveraged to assist in the decrease of disparities among cancer patients. Beacon will take advantage of these to accomplish this goal.
- Weakness: the integrity of data.

The SWOT analysis will then be used as the basis for a decision-support framework during the development of actionable plans. For example, in a scenario where “lack of knowledge on cancer centres” might be one of the identified threats, policymakers and providers might decide to develop plans geared toward raising the level of awareness and understanding of cancer facilities options, available infrastructure, and procedures. As yet another example, in a context where “multiple stakeholders” are identified as a Strength, creating collaborations that will bridge across different Cancer Centers would allow for capacity and capability sharing.

The SWOT analysis will be conducted in English with healthcare providers from different hospitals in different countries (e.g., Italy, Slovenia, Estonia, and Croatia).

### 2.8 Analysis plan

The sociodemographic data of participants will be analysed using descriptive statistics. Qualitative interview transcriptions will be first translated into English and then analysed with qualitative methods, through a specific software (e.g., qualitative analysis packages from the R software for statistical analysis). Specifically, thematic analysis will be performed to find patterns and themes related to disparities and inequalities in cancer outcomes (Vaismoradi et al., 2013). Each participant’s response will be read multiple times, to identify significant statements related to cancer disparities, assign codes to these statements, and, finally, form clusters of meaning from these significant statements into themes.

BEACON will act as a network facilitator among all stakeholders (i.e., patients, healthcare providers, researchers and policy makers), offering an opportunity to ensure appropriate cancer outcomes regardless of the region where patients might live. The dissemination of information regarding disparities across European countries, disseminated through a wide range of reports and Web applications will facilitate the tailoring of clinical practice guidelines. It will also provide resources for the subsequent adoption or implementation of strategies and planning related to cancer. Reports and graphs, both generic as well as customised, will be extensively generated using the oncology resource underlying the BEACON Wiki website. Also, since the information available within the The Beacon wiki website will be frequently curated, the reports and graphs will be constantly updated. Lastly, the ‘book down’ package in R will be used to generate printer-ready reports. In addition to the website, leaflets informing on the objectives and summarising the results of the project will be generated. The current premise is that while there is variability in cancer outcomes across EU countries, integration among stakeholders from different regions in the making of cancer plans can benefit both patients as well as the policymaking process.

## 3 Discussion

Considerable disparities exist between the quality of cancer outcomes, access to psycho-oncological support, awareness, and clinical outcomes in the cancer context across European communities, hospitals, regions, and countries (Gross et al., 2008; Hendren et al., 2010). Numerous factors at the level of the individual, the health care system and the broader social environment interact in complex ways to influence cancer disparities (Bertuccio et al., 2019). Thus, the BEACON project will explore those factors by asking patients, healthcare providers, researchers, and policy makers to communicate their needs, preferences and suggestions on how to address the cancer divide.

Expected results include potential reasons for cancer disparities from different stakeholders’ perspectives. Firstly, we believe we will capture different cancer patients’ difficulties in accessing care, managing potential delays and reasons for delays in diagnosis or treatment decisions, detecting which are the barriers to and facilitators of information-seeking behaviors they found, their decision-making processes, the unmet needs and the challenges in accessing psychological support. Several factors could contribute to cancer disparities, for example, geographic location and psychosocial aspects were found to affect the intention of searching for information and the access to sources, devices, and education (Jiang and Liu, 2020; Ferraris et al., 2023). Therefore, it is fundamental to involve cancer patients when addressing cancer disparities. Cancer patients are often unaware of the best healthcare professionals in proximity to their homes and often need better information and support (European Cancer Patient Coalition, 2015). The provision of appropriate information about cancer screening, diagnosis, treatment, and quality of life is crucial to help patients navigate the entire trajectory of cancer. However, in many cases, the quality of patient information provided is too complex and inaccessible to the average reader, leading to increased inequalities (Faller et al., 2016). Therefore, there is a strong need for an investigation of what are the core cancer patients’ needs and preferences regarding the quantity and quality of information and sources, as well as access to care, healthcare providers and financial aids (Vaccarella et al., 2023).

Secondly, healthcare providers could clarify their needs and the type of guidance when attempting to improve treatment quality and accessibility, making referrals, getting additional training, and in terms of resource sharing. Indeed, for healthcare providers, we expect to understand the ways they see integration between clinical practice and innovative research and the challenges that may arise in multidisciplinary teams (Denicoff et al., 2013). Moreover, our exploration seeks to unravel the intricacies healthcare providers face in communication dynamics, specifically delineating the challenges encountered when engaging with diverse patient demographics. Understanding these communication barriers is pivotal to tailor interventions that bridge gaps in understanding and care provision. Additionally, our focus extends to navigating the potential obstacles inherent within multidisciplinary team structures, recognizing the complexities and harnessing opportunities to optimize collaborative efforts. These multifaceted insights are anticipated to pave the way for a more cohesive and effective healthcare ecosystem, facilitating enhanced care provision and streamlined approaches to addressing cancer disparities.

Thirdly, for researchers, we expect to capture potential challenges in accessing existing datasets and sharing resources. This critical examination is pivotal to understanding and mitigating impediments that hinder the seamless flow of information essential for comprehensive research initiatives. In addition, researchers and healthcare providers could suggest what needs to be done for them in order to allow collaboration in planning more targeted and accessible clinical trials and what types of data would they be willing to learn about (Mills et al., 2006). By exploring these collaborative frameworks, we aim to uncover insights into the specific types of data researchers are keen to assimilate into their studies. Understanding their preferences and requirements concerning data acquisition becomes integral in shaping a more targeted research landscape that caters to the needs of both researchers and healthcare providers. This collaborative synergy is poised to pave the way for a more effective and efficient approach in conducting clinical trials, ultimately benefiting the advancement of cancer initiatives.

Lastly, policy makers, cooperating with researchers and healthcare providers, could indicate their requirements for a better allocation of resources and the implementation of guidelines and health policies with the scope of reducing the risk for cancer and increasing awareness among the target population (Bertuccio et al., 2019). Additionally, this cooperative initiative aims to develop the framework for the implementation of guidelines and health policies to reduce the risks associated with cancer and increase awareness among specific target populations.

Finally, findings collected in the focus group discussions will inform a subsequent Delphi study, and a SWOT analysis and the results will be used to increase information on cancer disparities and will guide the creation of a mobile application to inform patients, healthcare providers, researchers, and policy-makers with respect to available cancer treatment and care options across Europe, thereby promoting better access to personalised care. More precisely, to help patients find the best centres, providers in sharing resources and expertise, researchers in sharing data, and policy makers in aligning funding allocation with patients’ priorities.

## Ethics statement

The present qualitative study protocol received ethical approval from the Bioethics Committee of the University of Palermo on March 30th 2023 (approval number 141/2023), and informed consent was obtained from all subjects and/or their legal guardian(s). Moreover, the study is conducted in accordance with the Declaration of Helsinki Ethical Principles and Good Clinical Practices.

## Author contributions

GF, VC, DM, RG, and GP conceptualized the study, acquired funding, and planned the design and the methodology. GF, VC, and DM wrote the first draft of the manuscript. RG and GP reviewed and edited the manuscript. IK, DH, RP, and VG, participated in the conceptualization and will support the study procedure in different European countries. All authors contributed to the article and approved the submitted version.

## Supplementary material

SUPPLEMENTARY MATERIAL Focus Group Scheme
