# Supplementary material for: Addressing disparities in European cancer outcomes: a qualitative study Protocol of the BEACON project
Source: Front Psychol. 2024 Feb 26;15:1252832. doi: 10.3389/fpsyg.2024.1252832 (PMC10925749; doi:10.3389/fpsyg.2024.1252832)
Supplement: SUPPLEMENTARY MATERIAL — Focus Group Scheme [file Table_1.DOCX]

Supplementary Material

**FOCUS GROUP SCHEME**

**Introduction**

Good morning everyone,

thank you for your willingness to take part in this project. The Cancer Care Beacon project is an EU-funded project aimed at identifying and then reducing disparities in cancer care across Europe.

Let me introduce myself, I am XX, a psychologist at ....., and with me is ........ . Today I will be in charge of conducting this discussion while my colleague will be taking notes on what emerges not to lose any relevant information.

As you know, our meeting will last about 90 minutes. If necessary, we will have the opportunity to take breaks as needed. In order not to create confusion and to allow each of you to be able to express your thoughts, we would ask you to keep your microphones closed and ask for the floor by pressing the button to raise your hand or by writing it down in chat. I will then be the one to give you the floor. Please note that this meeting will be audio-video recorded. Only researchers on the project will have access to the recordings. These recordings will be eventually destroyed after being transcribed into a computer file when identifiable data like names and date of birth will be deleted and your responses will be recognized just by a code. More details regarding the data coding and processing appear on your information sheet. If you do not want that your opinions are being audio recorded, you should not sign the information sheet nor choose to participate in the focus group

In the past few days, you should have received a questionnaire; we ask if you have not yet filled it out, to do so in the next few days.

So today we propose to be able to discuss some issues by trying to think back to your experience. There is no right or wrong answer but it is crucial for us to know your opinion with respect to this specific moment.

We hope that the opinions we collect in this research will be useful to understand and address potential reasons for cancer disparities from different stakeholders’ perspectives.

For example, we believe we will capture different cancer patients’ difficulties in accessing care, potential delays and reasons for delays in diagnosis or treatment decisions, barriers to and facilitators of patients’ information-seeking behaviours and decision-making processes, unmet needs and challenges in accessing psychological support.

For healthcare providers, we expect to understand the ways they see integration between clinical practice and innovative research, difficulties in communicating with certain patients, and challenges in multidisciplinary teams.

For researchers, we expect to capture potential challenges in accessing existing datasets and sharing resources.

For policy-makers, we expect to understand their difficulties in allocating funding for cancer care.

Finally, the results will be used to increase information on cancer disparities and will guide the creation of a mobile application to inform patients, healthcare providers, researchers, and policy-makers with respect to available cancer treatment and care options across Europe, thereby promoting better access to personalized care.

Do you have any questions before starting?

Let's start the recording now.

**Short round of introduction**

In order to get to know each other better, we would like to propose a round in which everyone talks briefly (1-minute maximum each) about themselves (name, where you are from) and why you decided to participate in today's discussion.

**PATIENTS - Focus group scheme**

The discussion that we will have today is aimed at identifying reasons and themes that can contribute to cancer inequalities and disparities with a specific focus on obstacles related to prevention, screening, treatment, psychological support, and palliative care.

***Unmet needs and preferences in cancer care***

1. What have you found to be the most challenging (or difficult) for you since your diagnosis? For example, did you encounter any barriers to accessing treatments (e.g., difficulties in finding a good cancer centre, delays, lack of information on where to go etc)?
2. What services have been missing from your experience? Why (barriers and facilitators)? Which services would you have wished to be available for you?
3. Did you encounter any trouble in accessing or receiving cancer-related information?

If yes: Why was it difficult to find/receive information? What type of information was missing? On what aspects? For example, you struggled to understand when the doctor presented the treatment options to you, or you did not find what you were looking for, any information that you would not have wanted to receive or that you feel was unnecessary or contradictory etc.

If not: what helped you to reach relevant information? What information do you think is important to know? For example, the advantages and disadvantages of treatments, their duration, side effects, etc.

1. In what modality would you have liked to have received the information about the treatment(s)? For example, written text, video, brochure with concise but explanatory vignettes.

***Information seeking behaviours***

1. Did you search for more cancer information? Why?

If yes: was it easy? what information did you look for? Did you seek information from your primary care physician? Have you searched the Internet? On what portals? Did you seek information from someone who was/is involved in the same care journey as you?

If not: why did you not feel the need to look for more information?

***Decision-making processes***

1. How much involvement did you have in making decisions about your care and treatment?

If yes: what facilitated your inclusion in the decision-making process? Why?

If not: why you were not involved? What are the causes in your opinion?

***Psychological support***

1. Overall, have you felt adequately supported throughout your experiences with cancer and treatment? From who? In what you were supported/not supported?
2. Would you have wanted to receive more support?
3. Did you receive psychological support?

If yes: was it easy to access? Are you satisfied?

If not: Why not? Would you have wanted to receive psychological support?

**HEALTHCARE PROVIDERS - Focus group scheme**

The discussion that we will have today is aimed at identifying reasons and themes that can contribute to cancer inequalities and disparities with a specific focus on obstacles related to prevention, screening, treatment, psychological support, and palliative care.

***Unmet needs and preferences in cancer care***

1. What have you found to be the most challenging (or difficult) for you in dealing with patients?
2. What do you think are the biggest needs for cancer patients who are receiving treatment? Why?
3. What kind of help or supportive care do they need? Do you think it differs depending on the type of cancer they have?
4. What services/programs do you think are missing? Why are they missing? What would you like to see available to your patients with cancer?
5. What would facilitate offering more supportive care services? What are the barriers to providing these services?
6. What is the role of cancer screening in your practice? What would be an ideal scenario?
7. How do you perceive cancer disparities?

For example, do you perceive differences in public vs private care? What is the status of healthcare services in your city/country? Inequalities? Easy access to the best treatment?

1. How are your relationship and communication with your patients? And with your co-workers? Also, with other cancer centres (across and outside your country)? What hinders/facilitates communication with them?

***Information-seeking/providing***

1. Do you believe to have enough information when dealing with your patients? What would you need more?
2. What helps you (or not) to communicate with your patients? What helps you (or not) to communicate with your colleagues?

***Decision-making processes***

1. How much do you involve your patients in your treatment options? For example, do you use any techniques to discuss options with your patients? What can help you (or not) to involve more of your patients in the process?
2. What are the obstacles/facilitators of a shared decision process?

***Multidisciplinary care team***

1. Do you believe research and practice are well integrated in your work environment? How? What hinders/facilitates such integration?
2. Do you work in a multidisciplinary team?

If yes: is it a resource to you? Why?

If not: why? Would you desire to work in a multidisciplinary team/network?

1. Do you know/have access to clinical trials? What help you (or not) to have access to them?

**RESEARCHERS - Focus group scheme**

The discussion that we will have today is aimed at identifying reasons and themes that can contribute to cancer inequalities and disparities with a specific focus on obstacles related to prevention, screening, treatment, psychological support, and palliative care.

***Unmet needs and preferences***

1. Are you able to access existing datasets on cancer research?

If yes: What type? For example, disparity research literature? Are they publicly available? Do you need a proposal to access them? Cost to access?

If not: why? What makes them not available?

1. Do you have enough information on cancer registries in Europe? And access to them? Why? Which additional information you might need?
2. What help (or not) you to access research findings on cancer care? Why?
3. Are you able to share knowledge and resources in your research network? What helps you (or not) to share knowledge? Is it easy to collaborate with other experts in the field? Why?

**POLICY MAKERS - Focus group scheme**

The discussion that we will have today is aimed at identifying reasons and themes that can contribute to cancer inequalities and disparities with a specific focus on obstacles related to prevention, screening, treatment, psychological support, and palliative care.

1. Are you aware of any cancer disparities in your country? In other countries? Which ones?
2. Are you aware of any report/project against cancer disparities in your country? In other countries?

If yes: How is it? What works well (or not) in this project?

If not: what kind of project might be needed? Why?

1. What specific data and metrics do you believe are crucial to assess the capacities and capabilities of oncological centers in the European Union, in order to propose policies that improve cancer prevention, screening, treatments, and care?
2. What information gaps or challenges do you currently encounter when trying to understand the performance and quality of oncological centers in different EU member states? How could filling these gaps enhance your ability to propose effective policies?
3. Which areas in the cancer care pathway might need additional investment? Or reallocating funding? What can help (or not) to reallocate funding?
4. How is interdisciplinary collaboration in the cancer context? With whom do you have contact? For example, patients’ associations, healthcare providers, researchers etc. What hinders/facilitates such collaboration?
5. What is your plan for sustainability of projects in the cancer care? What would help (or not) sustainability plans?
